# Using Personalized Intervention Criteria in a Mobile Just-in-Time Adaptive Intervention for Increasing Physical Activity in University Students: Pilot Study

**DOI:** 10.2196/66750

**Published:** 2025-05-26

**Authors:** Mai Ikegaya, Jerome Clifford Foo, Taiga Murata, Kenta Oshima, Jinhyuk Kim

**Affiliations:** 1 Department of Informatics, Graduate School of Integrated Science and Technology, Shizuoka University, 3-5-1 Johoku, Chuo-ku, Hamamatsu, Shizuoka, 432-8011, Japan, 81 53-478-1526; 2 Department of Psychiatry, College of Health Sciences, University of Alberta, Edmonton, AB, Canada; 3 Neuroscience and Mental Health Institute, University of Alberta, Edmonton, AB, Canada; 4 Department of Genetic Epidemiology in Psychiatry, Central Institute of Mental Health, Medical Faculty Mannheim, University of Heidelberg, Mannheim, Germany; 5 Institute for Psychopharmacology, Central Institute of Mental Health, Medical Faculty Mannheim, University of Heidelberg, Mannheim, Germany

**Keywords:** physical activity, just-in-time adaptive intervention, mHealth, consumer-wearable activity trackers, multilevel model, activity tracker, wearable, behavioral change, effectiveness, activity monitor, activity monitoring, long-term intervention, chronic disease, prevention, treatment, mobile health, mobile phone, digital health, smartphone

## Abstract

**Background:**

While the health benefits of physical activity are well-known, adherence to regular physical activity remains a major challenge. Just-in-time adaptive intervention (JITAI) has been proposed as one method to increase physical activity by delivering an intervention at a time when individuals are more likely to make behavioral changes. However, most studies that have implemented JITAI have used uniform intervention criteria (UIC) across participants rather than personalized intervention criteria (PIC) for the individual.

**Objective:**

The objective of this paper was to examine the effectiveness of using JITAI implemented with PIC to increase physical activity.

**Methods:**

Healthy university students wore a wrist activity monitor for 2 weeks. Participants were divided into 2 groups, which received JITAI to promote physical activity according to either PIC or UIC. In the first week, the mean distance moved and sedentary time per hour for each participant were calculated to derive PIC. UIC was obtained from a 2-week study with a different sample (n=47) conducted under the same conditions. In the second week, JITAI prompts were sent every hour if both of the following criteria were met: the distance moved was shorter, and sedentary time was longer than PIC or UIC. Differences in changes in physical activity as a result of implementing interventions according to PIC and UIC were analyzed using multilevel models.

**Results:**

We analyzed data from 28 healthy university students (18‐23 y old, female n=12). Both PIC (*P*<.001) and UIC (*P*<.001) significantly increased physical activity in the first hour after JITAI was received. In that first hour, PIC increased physical activity more than UIC; more calories were burned (*P*=.02), more steps were taken (*P*=.007), and distance moved was increased (*P*=.003). However, over the course of the week, the use of JITAI did not significantly increase physical activity levels.

**Conclusions:**

Our results appear to suggest that PIC-based JITAI is more effective than UIC-based JITAI, consistent with the idea of a need for precision health approaches. Further research is needed to develop effective long-term intervention designs with sustainable effects.

## Introduction

The benefits of physical activity for prevention and management of chronic diseases and mortality have been well established [1-3]. To promote physical activity for better health, the World Health Organization (WHO) has recommended 150‐300 minutes of moderate-intensity aerobic physical activity per week, or 75‐150 minutes of vigorous-intensity aerobic physical activity throughout the week, or an equivalent combination of moderate- to vigorous-intensity activity [4]. However, a pooled analysis of 507 population-based surveys (n=5.7 million) found that 31.3% of adults worldwide do not meet this recommended amount of physical activity [5]. Low levels of daily physical activity have become a global health problem, and methods to increase physical activity using mobile health (mHealth) have started to be implemented [6-8].

mHealth is defined as health care services supported through the use of mobile devices such as mobile phones [9]. The aim of mHealth is to enhance users’ health and well-being through mobile devices and other wireless technologies by monitoring and analyzing their health status, providing support for disease prevention and treatment, and management and introduction of interventions for disease in everyday environments [10]. Previous studies incorporating mHealth interventions have shown beneficial effects in promoting health behaviors and support for behavioral change (eg, increasing physical activity [9,11,12]).

Just-in-time adaptive intervention (JITAI) is an intervention design that adapts the timing, type, and intensity of intervention support to changes in an individual’s status and behavior [13]. The goal is to provide support at the timing and in the context that the individual most needs and is most likely to accept it. JITAI has shown promising effects for improving physical activity in research settings. For example, van Dantzig et al [14] found that when they encouraged a 5-minute break after 60 minutes of continuous sitting, the amount of sedentary time decreased. Finkelstein et al [15] found that reminders promoting exercise sent when participants took 15 or fewer steps per hour significantly decreased sedentary behavior compared to when they did not receive reminders. Bond et al [16] found that 3-minute break reminders after 30 minutes of sitting decreased sedentary time and significantly increased the amount of time spent doing light physical activity compared to no reminder. Fiedler et al [17] found that delivering the prompt when the participant was inactive for more than 60 minutes significantly increased moderate to vigorous physical activity (MVPA). Mair et al [18] showed the feasibility of using personalized smartphone-delivered JITAI in elderly people. JITAI was tailored to the individual based on real time physical activity, daily physical activity goal, time of day, and weather conditions [18].

In most JITAI studies to date, however, the criteria for the intervention have been uniform (eg, 15 steps fewer per hour and sitting continuously for 30 or 60 minutes), and have not reflected the different activity patterns of the individual [12,18]. Uniform intervention criteria (UIC) do not accommodate varying levels of preexisting physical activity, and as such may not be the most effective way to promote uptake. In this study, we compared the effects of increasing activity in groups that received physical activity interventions using UIC versus groups that received an intervention using personalized intervention criteria (PIC) tailored to the individual’s activity patterns. We hypothesize that the intervention using PIC is more effective in increasing physical activity than the intervention using UIC.

## Methods

### Participants

A total of 31 healthy university students in Japan aged 18‐23 years old participated in the study. Healthy university students under 30 years of age were eligible to be included in the study. Participants who had current medical problems or were using medication that could influence physical activity were not eligible to participate. This prospective 2-week randomized comparative study took place between December 2022 and January 2023. The participants were randomly assigned into 2 groups in a 1:1 ratio by using a random number table (integers 0 and 1): PIC group, which received the intervention with personalized intervention criteria, and UIC group, which received the intervention with uniform intervention criteria. While a within-persons design was considered (both PIC and UIC in a single individual), due to the time limitations and potential mixing of effects of both types of intervention criteria, a between-persons design was used. Details are reported in the CONSORT-EHEALTH V 1.6.1 checklist (see Checklist 1).

### Ethical Considerations

The study was approved by the institutional review board at Shizuoka University, Japan (19‐8). Participants provided written informed consent after receiving a full explanation about the purpose of the study, methods, and precautions for participation in the study. Participants who completed the study received an Amazon gift card worth JP ¥5000 (approximately US $35).

All procedures performed in studies involving human participants were in accordance with the ethical standards of the institutional and national research committee. Participants provided written informed consent. All data used in this study were anonymized to protect participants’ privacy and ensure confidentiality.

### Assessments

#### Questionnaires

Participants completed a questionnaire before the 2-week study to collect their age, sex, height, weight, medical history, and drug use. Participants who had current medical problems or used medication were excluded from the study.

#### Activity Monitor

Physical activity was measured using a wrist-worn wearable activity monitor (Fitbit Inspire 2). The active monitor estimates sedentary time (minutes), lightly active time (minutes), fairly active time (minutes), very active time (minutes), calories (kcal), steps (n), and distance moved (m). Steps are calculated by the 3-axis accelerometer, while distance moved is automatically calculated based on the number of steps and the participant’s stride length. Time spent at different activity levels are also calculated by the activity monitor (sedentary time, lightly active time, fairly active time, and very active time). Activity monitor and participant’s smartphone were synchronized via Bluetooth by installing the Fitbit app on their smartphones, and activity data were recorded on the Fitbit server via the smartphone. The activity monitor was worn on the nondominant wrist at all times during the study period. Participants were instructed to remove the monitors when showering or bathing.

### Intervention

#### Intervention Criteria (PIC vs UIC)

The intervention criteria used were distance moved (m) and sedentary time (minutes). For distance moved, 1 SD lower than the mean (mean distance, SD) was used, and for sedentary time, 1 SD longer than the mean (mean sedentary time + SD) were used. The reason for extending the intervention criteria to include SD rather than using only the mean of physical activity was to ensure that interventions are not triggered when activity levels differed only slightly from the mean. That is, interventions might be too frequent and difficult to adhere to if the threshold was strictly set at the mean values.

Set points for interventions were determined differently for the 2 groups. PIC group received interventions based on individual activity data measured during week 1. The UIC group received interventions based on group data from a 2-week preliminary study conducted a year earlier using the same devices looking at physical activity in a sample of university students (n=47). The UIC were determined using a different sample because the timing of the study period was different for each participant; participants were recruited and participated during different dates so the UIC could not be calculated at the start of the intervention period for all participants.

#### Intervention Structure and Flow

**Figure 1.**
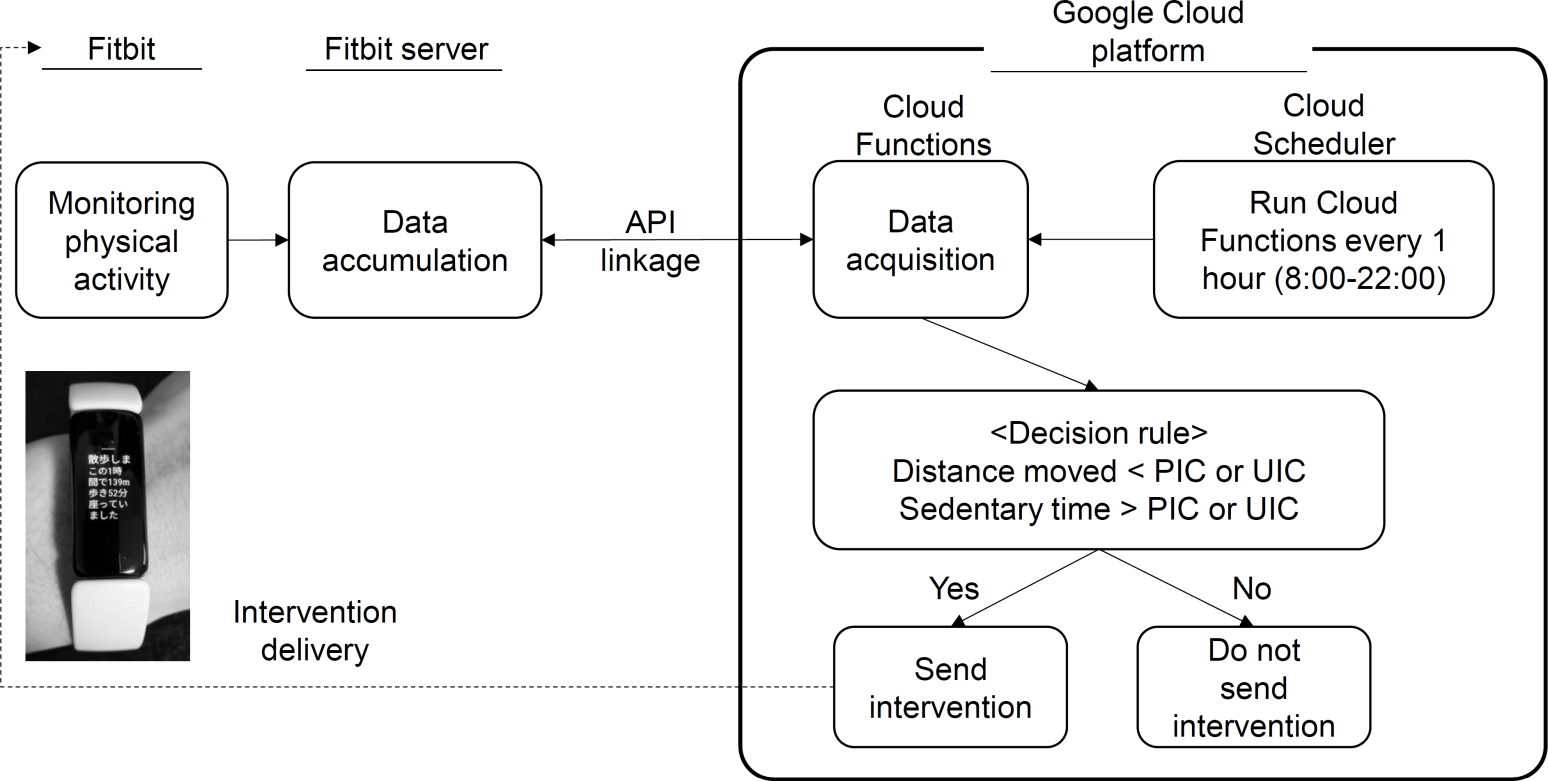
Intervention structure and flow. API: application programming interface; PIC: personalized intervention criteria; UIC: uniform intervention criteria.

Figure 1 shows the intervention structure and flow. The intervention was performed using the tools “Cloud Functions” and “Cloud Scheduler” of the Google Cloud platform via the Fitbit application programming interface (API). Cloud Functions is a serverless runtime environment used to build and connect cloud services and to run the process of retrieving physical activity data from activity monitor, calculating intervention criteria with the physical activity data and delivering feedback to participants as an intervention for increasing physical activity. Cloud Scheduler is a scheduling function for processing functions at a specific time, and was used to repeatedly run the process as needed. Cloud Scheduler was set up to trigger Cloud Functions at the 34-minute mark every hour (ie, 10:34 AM, 11:34 AM, 12:34 PM…) to evaluate the physical activity in the past hour (ie, 9:34 AM-10:33 AM, 10:34 AM-11:33 AM, 11:34 AM-12:33 PM…). Cloud Functions were used to retrieve 1-minute interval data from the Fitbit server through the Fitbit API. When both (1) “Distance moved” was less than the intervention criteria and (2) the “Sedentary time” was longer than the intervention criteria, participants were notified. Intervention decisions were based on data on distance moved and sedentary time in the past hour.

#### Intervention Timing

An intervention decision was made once per hour by the programmed Cloud Scheduler of the Google Cloud platform, and the intervention message was delivered when the intervention criteria met the predefined conditions (ie, UIC or PIC). The intervention schedule was run from 8:00 AM to 10:00 PM.

#### Intervention Contents

The intervention consisted of sending an email to the participant’s email address. The email included a message encouraging physical activity and stating the participant’s distance moved and sedentary time in the last hour. One message (in Japanese) was chosen at random each time from the following 5 messages: “Would you like to take a walk?” “Would you like to exercise?” “Move your body!” “Let’s exercise!” and “Let’s move a little!” The Fitbit app on the smartphone allowed email notifications, and when the email was received, the activity monitor vibrated, and the contents of the email were displayed on the Fitbit screen.

### Statistical Analysis

Analysis was performed using SAS 9.4 (SAS Institute Inc). The physical activity data acquired are longitudinal and repeatedly measured from individuals, and thus have a hierarchical structure. We used multilevel modeling, a widely used analytical method for such data structures [19]. We specified 2 different sets of models to examine the effectiveness of using JITAI implemented with PIC to increase physical activity. Model set A was specified to examine the effects of PIC or UIC on physical activity after the intervention. To examine the change in physical activity after 1 hour of intervention by different intervention criteria, the mean level of physical activity was estimated for the group using UIC and the group using PIC at the hour before and the hour after the intervention. The physical activity types estimated were sedentary time (minutes), lightly active time (minutes), fairly active time (minutes), very active time (minutes), calories (kcal), steps (n), and distance moved (m). We ran 7 different models, using each of these physical activity parameters as dependent variables. The intervention group (ie, PIC group and UIC group) and before or after intervention (ie, the hour before and the hour after the intervention) were used as independent variables.

### Model Set A

Level 1 model (within-participant):


(1) $$\begin{array}{rl} {Physical\;Activity}_{ti} & =\beta_{0i}+\beta_{1i}\\ \times \left({Before\;or\;After\;Intervention}_{ti}\right)+\varepsilon_{ti} \end{array}$$

Level 2 models (between-participants):


(2) $$\begin{array}{rl} \beta_{0i} & =\gamma_{00}+\gamma_{01}\times \left({Intervention\;Group}_{i}\right)\\ +\gamma_{02}\times \left({BMI}_{i}\right)+\gamma_{03}\times \left({Sex}_{i}\right)+\upsilon_{0i} \end{array}$$

(3) $$\begin{array}{c} \beta_{1i}=\gamma_{10}+\gamma_{11}\times \left(Intervention\;Group_{i}\right)+\upsilon_{1i} \end{array}$$

where “*Physical Activity_ti_*” indicates the dependent variable (sedentary time, lightly active time, fairly active time, very active time, calories, steps, or distance moved) at the *t*th recording for the *i*th participant; *β*_0i_ is participant i’s intercept; *β*_1i_ is participant i’s slope; *γ*_00_ is the average intercept across all participants; *γ*_01_, *γ*_02_, *γ*_03_, and *γ*_11_ are all participants’ slopes; *γ*_10_ is the slope of *β*_1i_; *ε*_ti_ is the within-individual residuals; *υ*_0i_ and *υ*_1i_ are the random effects of intercept and slope, respectively. The random or fixed effect of slope was determined by comparing Akaike information criterion (AIC) to find the best-fitting parameters for each. As an example, we have shown Equation 3 which has a random effect of slope (*υ*_1i_). To test the differences before or after intervention in mean level of physical activity, we added a categorical variable representing “*Before or After Intervention_ti_*” into the right-hand side of Equation 1. In addition, to test the difference in mean level of physical activity between PIC and UIC groups, we added a categorical variable representing “*Intervention Group_i_*” into the right-hand side of Equations 2 and 3. The slope (*γ*_11_) of an interaction term between “*Before or After Intervention_ti_*” and “*Intervention Group_i_*” represents the difference in physical activity for the group using UIC and the group using PIC at the hour before and the hour after the intervention in the combined multilevel model. In Equation 2, BMI and sex were adjusted for in the Level 2 (between-participants) models.

Model set B was specified to examine changes in physical activity from Week 1 (without intervention) to Week 2 (with intervention) associated with the different intervention criteria (UIC or PIC). A total of 7 separate models were specified using the hourly mean of each physical activity parameter (as above) as a dependent variable. Intervention group and week were specified as independent variables.

### Model Set B

Level 1 model (within-participant):


(4) $$\begin{array}{rl} {Physical\;Activity}_{ti} & =\beta_{0i}+\beta_{1i}\\ \times \left({Week\;1\;or\;Week\;2}_{ti}\right)+\varepsilon_{ti} \end{array}$$

Level 2 models (between-participants):


(5) $$\begin{array}{rl} \beta_{0i} & =\gamma_{00}+\gamma_{01}\times \left({Intervention\;Group}_{i}\right)\\ +\gamma_{02}\times \left({BMI}_{i}\right)+\gamma_{03}\times \left({Sex}_{i}\right)+\upsilon_{0i} \end{array}$$

(6) $$\begin{array}{c} \beta_{1i}=\gamma_{10}+\gamma_{11}\times \left(Intervention\;Group_{i}\right)+\upsilon_{1i} \end{array}$$

where “*Physical Activity_ti_*” indicates the dependent variable (sedentary time, lightly active time, fairly active time, very active time, calories, steps, or distance moved) at the *t*th recording for the *i*th participant; *β*_0i_ is participant i’s intercept; *β*_1i_ is participant i’s slope; *γ*_00_ is the average intercept across all participants; *γ*_01_, *γ*_02_, *γ*_03_, and *γ*_11_ are all participants’ slopes; *γ*_10_ is the slope of *β*_1i_; *ε*_ti_ is the within-individual residuals; *υ*_0i_ and *υ*_1i_ are the random effects of intercept and slope, respectively. The random or fixed effect of slope was determined by comparing AIC to find the best-fitting parameters for each. As an example, we have shown Equation 6 which has a random effect of slope (*υ*_1i_). To test the change in physical activity from Week 1 without intervention to Week 2 with intervention, we added a categorical variable representing “*Week 1 or Week 2_ti_*” into the right-hand side of Equation 4. In addition, to test the difference in mean level of physical activity between PIC and UIC groups, we added a categorical variable representing “*Intervention Group_i_*” into the right-hand side of Equations 5 and 6. In Equation 5, BMI and sex were adjusted for in the Level 2 (between-participants) models. In an effort to compare intervention effects more precisely, we used the hourly averaged physical activity to minimize variation due to factors such as sleep length and the time participants did not wear the activity monitor.

For all analyses, data were excluded when participants were sleeping and not wearing the activity monitor. A *P*<.05 was considered significant. The Tukey-Kramer method was used to adjust for multiple comparisons.

## Results

### Participant Characteristics

While 31 participants were recruited for the study, 3 participants did not participate for more than 4 days during their 2-week periods and were excluded from the analysis. There were no significant differences in characteristics between the intervention groups, except for in BMI (Table 1).

**Table 1.** Participant characteristics. Two-sample *t* tests were performed to compare the age, height, weight, and BMI between the PIC and UIC groups. *χ*^2^ tests were performed to compare the sex distribution across the two groups.

Characteristics	All participants (n=28)	PIC^a^ group (n=14)	UIC^b^ group (n=14)	*t* (*df*)	*χ*^2^ (*df*)	*P* value
Age (years), mean (SD)	20.6 (1.7)	20.6 (1.4)	20.6 (1.2)	0.00 (26)	—^c^	≥.99
Height (cm), mean (SD)	166.2 (7.3)	168.2 (6.9)	164.3 (7.2)	1.41 (26)	—	.17
Weight (kg), mean (SD)	54.8 (6.6)	53.3 (5.7)	56.3 (7.2)	−1.18 (26)	—	.25
BMI (kg/m^2^), mean (SD)	19.8 (1.9)	18.8 (1.1)	20.8 (2.0)	–3.24 (26)	—	.003
Sex				*—*	210.58 (1)	.45
Male, n (%)	16 (57)	9 (64)	7 (50)			
Female, n (%)	12 (43)	5 (36)	7 (50)			

a PIC: personalized intervention criteria.

b UIC: uniform intervention criteria.

c Not applicable.

The PIC group received interventions based on individual activity data measured during week 1. The UIC group received interventions based on group data from a 2-week preliminary study conducted a year earlier using the same devices looking at physical activity in a sample of university students (n=47; see Table 2).

**Table 2.** Participant intervention criteria.

ID	Distance moved (m)	Sedentary time (min)
PIC^a^1	151	50
PIC2	127	52
PIC3	125	52
PIC4	164	46
PIC5	263	41
PIC6	105	51
PIC7	93	55
PIC8	152	50
PIC9	148	50
PIC10	140	51
PIC11	39	56
PIC12	18	57
PIC13	57	55
PIC14	294	42
UIC^b^ (n=14)	139	52

a PIC: personalized intervention criteria.

b UIC: uniform intervention criteria.

**Figure 2.**
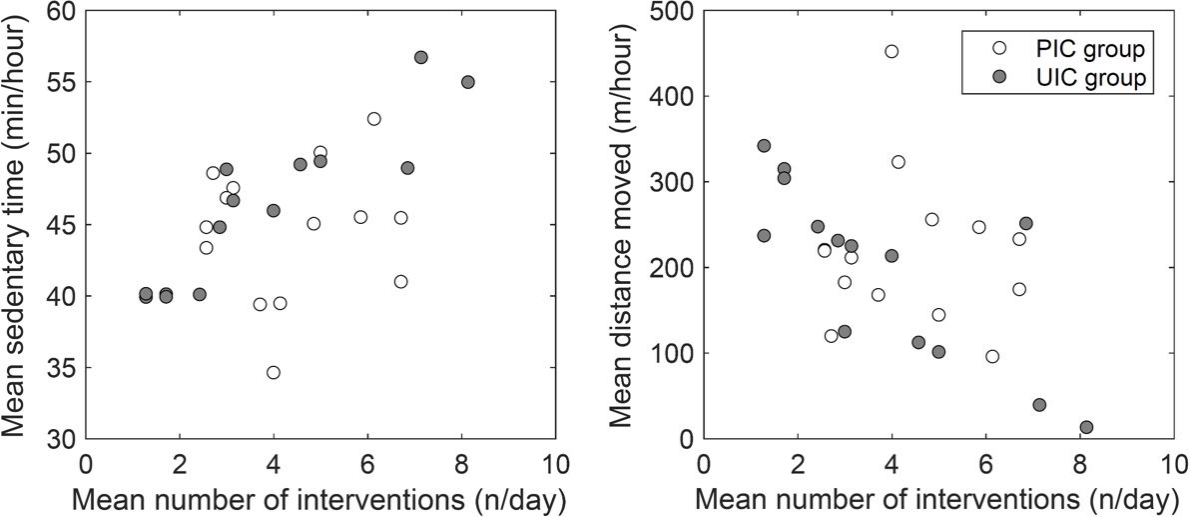
Mean number of interventions per day in accordance with physical activity level in personalized intervention criteria (PIC) versus uniform intervention criteria (UIC) group.

The mean number of interventions for the PIC group was 4.4 per day (SD 1.5; range 1.3‐8.1), and the mean number of interventions for the UIC group was 3.8 per day (SD 2.3; range 2.6‐6.7). No significant difference in the mean number of interventions was found between the PIC and UIC groups. However, some participants in the UIC group who had higher average levels of physical activity received a lower number of interventions (ie, a mean of 1‐2 interventions per day) to increase activity, while some participants who had lower average physical activity levels received a higher number of interventions (ie, a mean of 7‐8 interventions per day; see Figure 2).

### Model Set A: Effects of PIC and UIC on Physical Activity for the Hour Before and the Hour After the Intervention

In model set A, the best models for sedentary time and lightly active time used random intercepts and slopes, while the best models for fairly active time, very active time, calories, steps, and distance moved used random intercepts and fixed slopes. For both the PIC and UIC groups, sedentary time significantly decreased after the intervention (PIC group: before *B*=55.83, SE=0.54; after *B*=48.21, SE=1.07; *P*<.001 and UIC group: before *B*=57.11, SE=0.58; after *B*=49.88, SE=1.10; *P*<.001; see Table 3). The other 6 physical activity types (ie, lightly active time, fairly active time, very active time, calories, steps, and distance moved) significantly increased after the intervention in intervention groups (*P*<.001; see Table 3). See Table S1 in Multimedia Appendix 1 for detailed information.

**Table 3.** The change in physical activity 1 hour after the intervention as affected by differences in intervention criteria.

Physical activity	PIC^a^ group			UIC^b^ group		
	Before intervention	After intervention	*P* value	Before intervention	After intervention	*P* value
Sedentary time (min), mean (SE)	55.83 (0.54)	48.21 (1.07)	<.001	57.11 (0.58)	49.88 (1.10)	<.001
Lightly active time (min), mean (SE)	4.02 (0.53)	9.80 (0.94)	<.001	2.95 (0.56)	8.76 (0.97)	<.001
Fairly active time (min), mean (SE)	−0.04 (0.10)	0.86 (0.10)	<.001	0.08 (0.11)	0.65 (0.11)	.003
Very active time (min), mean (SE)	0.01 (0.12)	0.95 (0.12)	<.001	0.04 (0.13)	0.59 (0.13)	.01
Calories (kcal), mean (SE)	74.54 (3.08)	99.83 (3.08)	<.001	68.97 (3.24)	87.10 (3.24)	<.001
Steps (n), mean (SE)	66.35 (28.70)	403.35 (28.70)	<.001	63.80 (30.48)	285.67 (30.48)	<.001
Distance moved (m), mean (SE)	26.82 (12.52)	175.70 (12.52)	<.001	27.06 (13.31)	119.85 (13.31)	<.001

a PIC: personalized intervention criteria.

b UIC: uniform intervention criteria.

**Figure 3.**
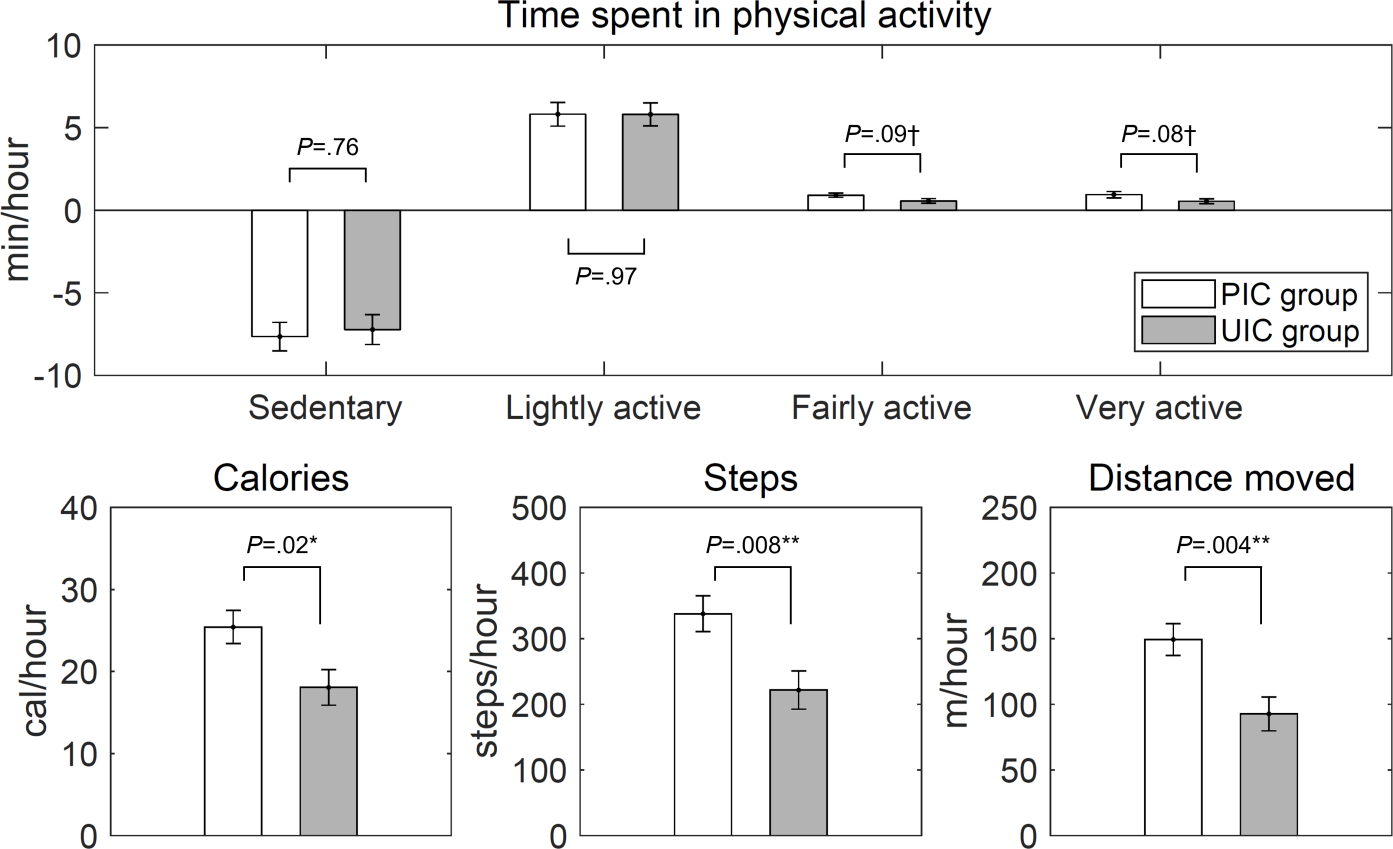
Changes in physical activity from the hour before to the hour after the intervention in personalized intervention criteria (PIC) versus uniform intervention criteria (UIC) group. The values represent time spent (min) doing physical activity (top), calories burned (kcal), steps taken (steps), or distance moved (m) per hour (bottom). ^*^P<.05 ^**^P<.01 ^†^*P*<.10.

Calories burned (PIC group: *B*=25.29, SE=2.03 and UIC group: *B*=18.13, SE=2.18; *P*=.02), Steps taken (PIC group: *B*=336.99, SE=27.45 and UIC group: *B*=221.88, SE=29.45; *P*=.008), and distance moved (PIC group: *B*=148.89, SE=12.19 and UIC group: *B*=92.79, SE=13.07; *P*=.004) showed significant differences in the increase in physical activity the hour before and after the intervention between the PIC group and the UIC group (Figure 3). Specifically, the number of calories burned per hour was estimated to increase by 7.16 kcal more in the PIC group than in the UIC group after the intervention. Steps taken increased by 115.11 steps and distance moved increased by 56.10 m in the PIC group compared with the UIC group. There were no significant differences in change observed between groups in the other activity types (see Table S1 in Multimedia Appendix 1 for details).

### Model Set B: Effects of PIC and UIC on Physical Activity From Week 1 Without Intervention to Week 2 With Intervention

In model set B, the best models for lightly active time, fairly active time, calories, steps, and distance moved used random intercepts and slopes, while the best models for sedentary time and very active time used random intercepts and fixed slopes. In both PIC and UIC groups, there were no significant changes in physical activity (all parameters) between week 1 and week 2 (see Table 4). See Table S2 in Multimedia Appendix 1 for detailed information.

**Table 4.** The change in physical activity from week 1 without intervention to week 2 with intervention as affected by differences in intervention criteria.

Physical activity	PIC group			UIC group		
	Week 1	Week 2	*P* value	Week 1	Week 2	*P* value
Sedentary time (min), mean (SE)	43.01 (1.36)	42.26 (1.36)	.55	44.82 (1.36)	45.38 (1.36)	.76
Lightly active time (min), mean (SE)	14.01 (1.19)	14.52 (1.12)	.92	12.81 (1.19)	11.94 (1.12)	.69
Fairly active time (min), mean (SE)	1.48 (0.21)	1.75 (0.29)	.67	1.35 (0.21)	1.70 (0.30)	.50
Very active time (min), mean (SE)	1.44 (0.19)	1.41 (0.19)	≥.99	1.06 (0.19)	1.02 (0.19)	≥.99
Calories (kcal), mean (SE)	116.91 (4.36)	119.06 (5.81)	.94	106.99 (4.34)	105.32 (5.80)	.97
Steps (steps), mean (SE)	590.46 (58.36)	564.56 (62.62)	.94	525.15 (58.00)	497.16 (62.50)	.93
Distance moved (m), mean (SE)	257.31 (26.14)	245.49 (27.81)	.93	230.32 (25.98)	214.77 (27.75)	.85

## Discussion

### Principal Findings

In this study, we examined the differences between in the impact of interventions based on personalized intervention criteria and uniform intervention criteria on physical activity in daily life. We found that in the first hour after JITAI was delivered, physical activity in the PIC group increased more than in the UIC group. However, the use of JITAI (both criteria) did not significantly increase physical activity over the whole study period.

There was a significant difference in increase in calories burned, steps taken, and distance moved between the PIC and UIC groups. This indicates that interventions based on PIC were more effective, suggesting that interventions to promote physical activity need to use individually developed intervention criteria based on physical activity in daily life. It has been reported that low feasibility of intervention suggestions is likely to lead to dissatisfaction [20,21]. It is possible that the setting of individually optimized intervention criteria that presented highly feasible goals (eg, proper number of interventions; see Figure 2) based on their physical activity level may have made it easier for them to change their behavior. Further studies need to identify the optimal number of interventions per day to promote physical activity.

Interestingly, in contrast to previous studies, we did not observe differences in overall physical activity between the first week without intervention and the week with intervention in either group. This may be due to the length of the intervention period used in this study. A previous study using UIC showed found an increase in physical activity after 21 days of intervention [16], while another study that sent contextually tailored intervention messages for 6 weeks showed an increase in the number of steps, although the increase diminished over time [22]. Taken together, it is possible that a longer intervention period is needed to effectively promote increased physical activity. A possible additional factor explaining this was that all but one participant had never worn activity monitors before, and simply wearing the monitors during the first week might have affected their baseline activity levels [23]. Future studies may need to set a burn-in period to precisely identify the baseline of physical activity before starting intervention.

This study had several limitations. Participants were university students, and the number of participants was small (n=28). Considering the sample size and the limited number of personal characteristics available to include as covariates (ie, age, BMI, and sex), it is possible that group differences (PIC vs UIC) may be related to other hidden third variables. Further studies need to examine the effectiveness of JITAI using PIC in larger samples with more personal characteristics, especially for participants who have low average levels of physical activity (eg, obese and elderly people who need more personalized intervention criteria). In addition, the variables of physical activity (ie, sedentary time and distance moved) we used for intervention were limited and in the future, more criteria could be used to determine thresholds to trigger interventions, and more sophisticated approaches to determine intervention decisions (eg, machine learning classification) could be used with additional physical activity features. A technical limitation was that the time of the intervention decision could not be randomized and was fixed. In future JITAI research, a finer grained approach ensuring that it is possible to intervene at the timing when the intervention criteria are met might lead to more optimal timing of the intervention. Finally, this study was limited to 2 weeks in duration and did not include any follow-up. Programs for increasing physical activity require the promotion of long-term behavioral changes. Further studies need to consider maintenance of benefits and sustainability of programs over longer periods of time.

### Conclusion

We found that personalized intervention criteria were more effective than uniform intervention criteria in increasing physical activity levels, supporting the idea that precision health approaches tailored to the individual may lead to beneficial effects on health. The findings of this pilot study warrant further investigation; JITAI PIC programs need to be assessed over longer durations and in different populations.

## Supplementary material

10.2196/66750 Multimedia Appendix 1 Supplementary tables providing detailed information on the effects of intervention criteria on physical activity.

10.2196/66750 Checklist 1 CONSORT-EHEALTH checklist. CONSORT: Consolidated Standards of Reporting Trails.

## Abbreviations

AIC: Akaike information criterion
API: application programming interface
JITAI: just-in-time adaptive intervention
mHealth: mobile health
MVPA: moderate to vigorous intensity physical activity
PIC: personalized intervention criteria
UIC: uniform intervention criteria
WHO: World Health Organization
