# Supplementary material for: Using Personalized Intervention Criteria in a Mobile Just-in-Time Adaptive Intervention for Increasing Physical Activity in University Students: Pilot Study
Source: JMIR Hum Factors. 2025 May 26;12:e66750. doi: 10.2196/66750 (PMC12129369; doi:10.2196/66750)
Supplement: Multimedia Appendix 1 [file humanfactors-v12-e66750-s001.docx]

*Supplementary material to:*

**Using Personalized Intervention Criteria in a Mobile Just-in-Time Adaptive Intervention for Increasing Physical Activity in University Students: A Pilot Study**

Table S1. The change in physical activity one hour after intervention as affected by differences in intervention criteria (Model Set A) (PIC group)

|  | PIC group | | | | | | | |
| --- | --- | --- | --- | --- | --- | --- | --- | --- |
|  | Before  Intervention | After  Intervention | Coefficient | SE | *df* | *t* | *P* | *Adjusted P* |
| Sedentary time (min) | 55.83 (0.54) | 48.21 (1.07) | 7.62 | 0.86 | 26 | 8.88 | <.001^***^ | <.001^***^ |
| Lightly Active time (min) | 4.02 (0.53) | 9.80 (0.94) | -5.77 | 0.68 | 26 | -8.44 | <.001^***^ | <.001^***^ |
| Fairly Active time (min) | -0.04 (0.10) | 0.86 (0.10) | -0.91 | 0.13 | 26 | -6.76 | <.001^***^ | <.001^***^ |
| Very Active time (min) | 0.01 (0.12) | 0.95 (0.12) | -0.94 | 0.15 | 26 | -6.34 | <.001^***^ | <.001^***^ |
| Calories (kcal) | 74.54 (3.08) | 99.83 (3.08) | -25.29 | 2.03 | 26 | -12.44 | <.001^***^ | <.001^***^ |
| Steps (steps) | 66.35 (28.70) | 403.35 (28.70) | -336.99 | 27.45 | 26 | -12.28 | <.001^***^ | <.001^***^ |
| Distance moved (m) | 26.82 (12.52) | 175.70 (12.52) | -148.89 | 12.19 | 26 | -12.22 | <.001^***^ | <.001^***^ |

Note. The values represent time spent (min), Calories burned (kcal), Steps taken (steps), or Distance moved (m) per hour. Standard Error (SE). Degree of Freedom (*df*). The Tukey-Kramer method was used to adjust for multiple comparisons. ^*^ *P*<.05 ^**^ *P*<.01 ^***^ *P*<.001

Table S1. The change in physical activity one hour after intervention as affected by differences in intervention criteria (Model Set A) (UIC group)

|  | UIC group | | | | | | | |
| --- | --- | --- | --- | --- | --- | --- | --- | --- |
|  | Before  Intervention | After  Intervention | Coefficient | SE | *df* | *t* | *P* | *Adjusted P* |
| Sedentary time (min) | 57.11 (0.58) | 49.88 (1.10) | 7.23 | 0.89 | 26 | 8.08 | <.001^***^ | <.001^***^ |
| Lightly Active time (min) | 2.95 (0.56) | 8.76 (0.97) | -5.81 | 0.71 | 26 | -8.13 | <.001^***^ | <.001^***^ |
| Fairly Active time (min) | 0.08 (0.11) | 0.65 (0.11) | -0.56 | 0.14 | 26 | -3.91 | .001^**^ | .003^**^ |
| Very Active time (min) | 0.04 (0.13) | 0.59 (0.13) | -0.55 | 0.16 | 26 | -3.44 | .002^**^ | .010^*^ |
| Calories (kcal) | 68.97 (3.24) | 87.10 (3.24) | -18.13 | 2.18 | 26 | -8.32 | <.001^***^ | <.001^***^ |
| Steps (steps) | 63.80 (30.48) | 285.67 (30.48) | -221.88 | 29.45 | 26 | -7.54 | <.001^***^ | <.001^***^ |
| Distance moved (m) | 27.06 (13.31) | 119.85 (13.31) | -92.79 | 13.07 | 26 | -7.10 | <.001^***^ | <.001^***^ |

Note. The values represent time spent (min), Calories burned (kcal), Steps taken (steps), or Distance moved (m) per hour. Standard Error (SE). Degree of Freedom (*df*). The Tukey-Kramer method was used to adjust for multiple comparisons. ^*^ *P*<.05 ^**^ *P*<.01 ^***^ *P*<.001

Table S1-1. The change in physical activity one hour after intervention as affected by differences in intervention criteria (Model Set A) (Sedentary time)

|  | Coefficient | SE | *df* | *t* | *P* |
| --- | --- | --- | --- | --- | --- |
| Intercept ($\gamma_{00}$) | 52.08 | 4.80 | 24 | 10.85 | <.001^***^ |
| Before or After Intervention ($\gamma_{10}$) | 7.62 | 0.86 | 26 | 8.88 | <.001^***^ |
| Intervention Group ($\gamma_{01}$) | 1.67 | 1.58 | 24 | 1.06 | .300 |
| Before or After Intervention × Intervention Group ($\gamma_{11}$) | -0.38 | 1.24 | 26 | -0.31 | .759 |
| BMI ($\gamma_{02}$) | -0.17 | 0.25 | 24 | -0.71 | .487 |
| Sex ($\gamma_{03}$) | -1.01 | 0.74 | 24 | -1.36 | .185 |

Note. Before or After Intervention was coded as a dummy variable (Before Intervention = 0, After Intervention = 1). Intervention Group was coded as a dummy variable (UIC = 0, PIC = 1). Standard Error (SE). Degree of Freedom (*df*). ^*^ *P*<.05 ^**^ *P*<.01 ^***^ *P*<.001.

Table S1-2. The change in physical activity one hour after intervention as affected by differences in intervention criteria (Model Set A) (Lightly Activity time)

|  | Coefficient | SE | *df* | *t* | *P* |
| --- | --- | --- | --- | --- | --- |
| Intercept ($\gamma_{00}$) | 6.72 | 4.55 | 24 | 1.48 | .153 |
| Before or After Intervention ($\gamma_{10}$) | -5.77 | 0.68 | 26 | -8.44 | <.001^***^ |
| Intervention Group ($\gamma_{01}$) | -1.04 | 1.40 | 24 | -0.74 | .465 |
| Before or After Intervention ×　Intervention Group ($\gamma_{11}$) | -0.04 | 0.99 | 26 | -0.04 | .971 |
| BMI ($\gamma_{02}$) | 0.14 | 0.23 | 24 | 0.58 | .568 |
| Sex ($\gamma_{03}$) | 0.93 | 0.71 | 24 | 1.31 | .202 |

Note. Before or After Intervention was coded as a dummy variable (Before Intervention = 0, After Intervention = 1). Intervention Group was coded as a dummy variable (UIC = 0, PIC = 1). Standard Error (SE). Degree of Freedom (*df*). ^*^ *P*<.05 ^**^ *P*<.01 ^***^ *P*<.001

Table S1-3. The change in physical activity one hour after intervention as affected by differences in intervention criteria (Model Set A) (Fairly Activity time)

|  | Coefficient | SE | *df* | *t* | *P* |
| --- | --- | --- | --- | --- | --- |
| Intercept $\gamma_{00}$) | 2.31 | 0.71 | 24 | 3.26 | .003^**^ |
| Before or After Intervention ($\gamma_{10}$) | -0.91 | 0.13 | 26 | -6.76 | <.001^***^ |
| Intervention Group ($\gamma_{01}$) | -0.22 | 0.16 | 24 | -1.37 | .184 |
| Before or After Intervention × Intervention Group ($\gamma_{11}$) | 0.34 | 0.20 | 26 | 1.75 | .091 |
| BMI ($\gamma_{02}$) | -0.07 | 0.04 | 24 | -1.90 | .069 |
| Sex ($\gamma_{03}$) | -0.17 | 0.11 | 24 | -1.56 | .132 |

Note. Before or After Intervention was coded as a dummy variable (Before Intervention = 0, After Intervention = 1). Intervention Group was coded as a dummy variable (UIC = 0, PIC = 1). Standard Error (SE). Degree of Freedom (*df*). ^*^ *P*<.05 ^**^ *P*<.01 ^***^ *P*<.001

Table S1-4. The change in physical activity one hour after intervention as affected by differences in intervention criteria (Model Set A) (Very Activity time)

|  | Coefficient | SE | *df* | *t* | *P* |
| --- | --- | --- | --- | --- | --- |
| Intercept ($\gamma_{00}$) | 0.62 | 0.89 | 24 | 0.70 | .494 |
| Before or After Intervention ($\gamma_{10}$) | -0.94 | 0.15 | 26 | -6.34 | <.001^***^ |
| Intervention Group ($\gamma_{01}$) | -0.36 | 0.19 | 24 | -1.89 | .070 |
| Before or After Intervention × Intervention Group ($\gamma_{11}$) | 0.39 | 0.22 | 26 | 1.81 | .083 |
| BMI ($\gamma_{02}$) | 0.02 | 0.05 | 24 | 0.47 | .640 |
| Sex ($\gamma_{03}$) | -0.24 | 0.14 | 24 | -1.76 | .091 |

Note. Before or After Intervention was coded as a dummy variable (Before Intervention = 0, After Intervention = 1). Intervention Group was coded as a dummy variable (UIC = 0, PIC = 1). Standard Error (SE). Degree of Freedom (*df*). ^*^ *P*<.05 ^**^ *P*<.01 ^***^ *P*<.001

Table S1-5. The change in physical activity one hour after intervention as affected by differences in intervention criteria (Model Set A) (Calories)

|  | Coefficient | SE | *df* | *t* | *P* |
| --- | --- | --- | --- | --- | --- |
| Intercept ($\gamma_{00}$) | 45.18 | 24.05 | 24 | 1.88 | .073 |
| Before or After Intervention ($\gamma_{10}$) | -25.29 | 2.03 | 26 | -12.44 | <.001^***^ |
| Intervention Group ($\gamma_{01}$) | -12.73 | 4.80 | 24 | -2.65 | .014^*^ |
| Before or After Intervention × Intervention Group ($\gamma_{11}$) | 7.16 | 2.98 | 26 | 2.40 | .023^*^ |
| BMI ($\gamma_{02}$) | 3.18 | 1.26 | 24 | 2.52 | .019^*^ |
| Sex ($\gamma_{03}$) | -18.35 | 3.93 | 24 | -4.67 | <.001^***^ |

Note. Before or After Intervention was coded as a dummy variable (Before Intervention = 0, After Intervention = 1). Intervention Group was coded as a dummy variable (UIC = 0, PIC = 1). Standard Error (SE). Degree of Freedom (*df*). ^*^ *P*<.05 ^**^ *P*<.01 ^***^ *P*<.001

Table S1-6. The change in physical activity one hour after intervention as affected by differences in intervention criteria (Model Set A) (Steps)

|  | Coefficient | SE | *df* | *t* | *P* |
| --- | --- | --- | --- | --- | --- |
| Intercept ($\gamma_{00}$) | 410.71 | 216.64 | 24 | 1.90 | .070 |
| Before or After Intervention ($\gamma_{10}$) | -336.99 | 27.45 | 26 | -12.28 | <.001^***^ |
| Intervention Group ($\gamma_{01}$) | -117.67 | 44.38 | 24 | -2.65 | .014^*^ |
| Before or After Intervention × Intervention Group ($\gamma_{11}$) | 115.11 | 40.26 | 26 | 2.86 | .008^**^ |
| BMI ($\gamma_{02}$) | -0.01 | 11.31 | 24 | -0.00 | .999 |
| Sex ($\gamma_{03}$) | -16.63 | 34.54 | 24 | -0.48 | .635 |

Note. Before or After Intervention was coded as a dummy variable (Before Intervention = 0, After Intervention = 1). Intervention Group was coded as a dummy variable (UIC = 0, PIC = 1). Standard Error (SE). Degree of Freedom (*df*). ^*^ *P*<.05 ^**^ *P*<.01 ^***^ *P*<.001

Table S1-7. The change in physical activity one hour after intervention as affected by differences in intervention criteria (Model Set A) (Distance moved)

|  | Coefficient | SE | *df* | *t* | *P* |
| --- | --- | --- | --- | --- | --- |
| Intercept ($\gamma_{00}$) | 194.47 | 94.26 | 24 | 2.06 | .050 |
| Before or After Intervention ($\gamma_{10}$) | -148.89 | 12.19 | 26 | -12.22 | <.001^***^ |
| Intervention Group ($\gamma_{01}$) | -55.86 | 19.36 | 24 | -2.89 | .008^**^ |
| Before or After Intervention × Intervention Group ($\gamma_{11}$) | 56.10 | 17.87 | 26 | 3.14 | .004^**^ |
| BMI ($\gamma_{02}$) | -0.60 | 4.92 | 24 | -0.12 | .904 |
| Sex ($\gamma_{03}$) | -16.12 | 15.00 | 24 | -1.07 | .293 |

Note. Before or After Intervention was coded as a dummy variable (Before Intervention = 0, After Intervention = 1). Intervention Group was coded as a dummy variable (UIC = 0, PIC = 1). Standard Error (SE). Degree of Freedom (*df*). ^*^ *P*<.05 ^**^ *P*<.01 ^***^ *P*<.001

Table S2. The change in physical activity from Week 1 without intervention to Week 2 with intervention as affected by differences in intervention criteria (Model Set B) (PIC group)

|  | PIC group | | | | | | | |
| --- | --- | --- | --- | --- | --- | --- | --- | --- |
|  | Week 1 | Week 2 | Coefficient | SE | *df* | *t* | *P* | *Adjusted P* |
| Sedentary time (min) | 43.01 (1.36) | 42.26 (1.36) | 0.75 | 0.57 | 26 | 1.33 | .196 | .554 |
| Lightly Active time (min) | 14.01 (1.19) | 14.52 (1.12) | -0.51 | 0.79 | 26 | -0.65 | .524 | .916 |
| Fairly Active time (min) | 1.48 (0.21) | 1.75 (0.29) | -0.27 | 0.24 | 26 | -1.14 | .267 | .672 |
| Very Active time (min) | 1.44 (0.19) | 1.41 (0.19) | 0.03 | 0.17 | 26 | 0.21 | .837 | .997 |
| Calories (kcal) | 116.91 (4.36) | 119.06 (5.81) | -2.15 | 3.77 | 26 | -0.57 | .574 | .940 |
| Steps (steps) | 590.46 (58.36) | 564.56 (62.62) | 25.90 | 45.22 | 26 | 0.57 | .572 | .939 |
| Distance moved (m) | 257.31 (26.14) | 245.49 (27.81) | 11.83 | 19.36 | 26 | 0.61 | .547 | .928 |

Note. The values represent time spent (min), Calories burned (kcal), Steps taken (steps), or Distance moved (m) per hour. Standard Error (SE). Degree of Freedom (*df*). The Tukey-Kramer method was used to adjust for multiple comparisons. ^*^ *P*<.05 ^**^ *P*<.01 ^***^ *P*<.001

Table S2. The change in physical activity from Week 1 without intervention to Week 2 with intervention as affected by differences in intervention criteria (Model Set B) (UIC group)

|  | UIC group | | | | | | | |
| --- | --- | --- | --- | --- | --- | --- | --- | --- |
|  | Week 1 | Week 2 | Coefficient | SE | *df* | *t* | *P* | *Adjusted P* |
| Sedentary time (min) | 44.82 (1.36) | 45.38 (1.36) | -0.56 | 0.57 | 26 | -0.98 | .335 | .760 |
| Lightly Active time (min) | 12.81 (1.19) | 11.94 (1.12) | 0.87 | 0.79 | 26 | 1.10 | .280 | .691 |
| Fairly Active time (min) | 1.35 (0.21) | 1.70 (0.30) | -0.34 | 0.24 | 26 | -1.42 | .167 | .498 |
| Very Active time (min) | 1.06 (0.19) | 1.02 (0.19) | 0.04 | 0.17 | 26 | 0.23 | .821 | .996 |
| Calories (kcal) | 106.99 (4.34) | 105.32 (5.80) | 1.67 | 3.77 | 26 | 0.44 | .662 | .971 |
| Steps (steps) | 525.15 (58.00) | 497.16 (62.50) | 27.99 | 45.22 | 26 | 0.62 | .541 | .925 |
| Distance moved (m) | 230.32 (25.98) | 214.77 (27.75) | 15.55 | 19.36 | 26 | 0.80 | .429 | .852 |

Note. The values represent time spent (min), Calories burned (kcal), Steps taken (steps), or Distance moved (m) per hour. Standard Error (SE). Degree of Freedom (*df*). The Tukey-Kramer method was used to adjust for multiple comparisons. ^*^ *P*<.05 ^**^ *P*<.01 ^***^ *P*<.001

Table S2-1. The change in physical activity from Week 1 without intervention to Week 2 with intervention as affected by differences in intervention criteria (Model Set B) (Sedentary time)

|  | Coefficient | SE | *df* | *t* | *P* |
| --- | --- | --- | --- | --- | --- |
| Intercept ($\gamma_{00}$) | 52.65 | 10.41 | 24 | 5.06 | <.001^***^ |
| Week 1 or Week 2 ($\gamma_{10}$) | 0.75 | 0.57 | 26 | 1.33 | .196 |
| Intervention Group ($\gamma_{01}$) | 3.12 | 2.09 | 24 | 1.49 | .149 |
| Week 1 or Week 2 × Intervention Group ($\gamma_{11}$) | -1.31 | 0.80 | 26 | -1.63 | .114 |
| BMI ($\gamma_{02}$) | -0.55 | 0.55 | 24 | -1.00 | .326 |
| Sex ($\gamma_{03}$) | 1.06 | 1.74 | 24 | 0.61 | .547 |

Note. Week 1 or Week 2 was coded as a dummy variable (Week 1 = 0, Week 2 = 1). Intervention Group was coded as a dummy variable (UIC = 0, PIC = 1). Standard Error (SE). Degree of Freedom (*df*). ^*^ *P*<.05 ^**^ *P*<.01 ^***^ *P*<.001.

Table S2-2. The change in physical activity from Week 1 without intervention to Week 2 with intervention as affected by differences in intervention criteria (Model Set B) (Light Activity time)

|  | Coefficient | SE | *df* | *t* | *P* |
| --- | --- | --- | --- | --- | --- |
| Intercept ($\gamma_{00}$) | 8.82 | 8.44 | 24 | 1.04 | .307 |
| Week 1 or Week 2 ($\gamma_{10}$) | -0.51 | 0.79 | 26 | -0.65 | .524 |
| Intervention Group ($\gamma_{01}$) | -2.58 | 1.71 | 24 | -1.51 | .144 |
| Week 1 or Week 2 × Intervention Group ($\gamma_{11}$) | 1.39 | 1.12 | 26 | 1.24 | .227 |
| BMI ($\gamma_{02}$) | 0.28 | 0.44 | 24 | 0.63 | .535 |
| Sex ($\gamma_{03}$) | 0.46 | 1.41 | 24 | 0.33 | .747 |

Note. Week 1 or Week 2 was coded as a dummy variable (Week 1 = 0, Week 2 = 1). Intervention Group was coded as a dummy variable (UIC = 0, PIC = 1). Standard Error (SE). Degree of Freedom (*df*). ^*^ *P*<.05 ^**^ *P*<.01 ^***^ *P*<.001

Table S2-3. The change in physical activity from Week 1 without intervention to Week 2 with intervention as affected by differences in intervention criteria (Model Set B) (Fairly Activity time)

|  | Coefficient | SE | *df* | *t* | *P* |
| --- | --- | --- | --- | --- | --- |
| Intercept ($\gamma_{00}$) | 1.87 | 1.60 | 24 | 1.17 | .254 |
| Week 1 or Week 2 ($\gamma_{10}$) | -0.27 | 0.24 | 26 | -1.14 | .267 |
| Intervention Group ($\gamma_{01}$) | -0.06 | 0.44 | 24 | -0.13 | .900 |
| Week 1 or Week 2 × Intervention Group ($\gamma_{11}$) | -0.07 | 0.34 | 26 | -0.20 | .841 |
| BMI ($\gamma_{02}$) | 0.01 | 0.08 | 24 | 0.15 | .879 |
| Sex ($\gamma_{03}$) | -0.87 | 0.27 | 24 | -3.25 | .003^**^ |

Note. Week 1 or Week 2 was coded as a dummy variable (Week 1 = 0, Week 2 = 1). Intervention Group was coded as a dummy variable (UIC = 0, PIC = 1). Standard Error (SE). Degree of Freedom (*df*). ^*^ *P*<.05 ^**^ *P*<.01 ^***^ *P*<.001

Table S2-4. The change in physical activity from Week 1 without intervention to Week 2 with intervention as affected by differences in intervention criteria (Model Set B) (Very Activity time)

|  | Coefficient | SE | *df* | *t* | *P* |
| --- | --- | --- | --- | --- | --- |
| Intercept ($\gamma_{00}$) | -2.52 | 1.31 | 24 | -1.92 | .067 |
| Week 1 or Week 2 ($\gamma_{10}$) | 0.03 | 0.17 | 26 | 0.21 | .837 |
| Intervention Group ($\gamma_{01}$) | -0.39 | 0.29 | 24 | -1.36 | .186 |
| Week 1 or Week 2 × Intervention Group ($\gamma_{11}$) | 0.00 | 0.23 | 26 | 0.01 | .988 |
| BMI ($\gamma_{02}$) | 0.21 | 0.07 | 24 | 3.13 | .005^**^ |
| Sex ($\gamma_{03}$) | -0.77 | 0.22 | 24 | -3.50 | .002^**^ |

Note. Week 1 or Week 2 was coded as a dummy variable (Week 1 = 0, Week 2 = 1). Intervention Group was coded as a dummy variable (UIC = 0, PIC = 1). Standard Error (SE). Degree of Freedom (*df*). ^*^ *P*<.05 ^**^ *P*<.01 ^***^ *P*<.001

Table S2-5. The change in physical activity from Week 1 without intervention to Week 2 with intervention as affected by differences in intervention criteria (Model Set B) (Calories)

|  | Coefficient | SE | *df* | *t* | *P* |
| --- | --- | --- | --- | --- | --- |
| Intercept ($\gamma_{00}$) | 39.85 | 34.13 | 24 | 1.17 | .254 |
| Week 1 or Week 2 ($\gamma_{10}$) | -2.15 | 3.77 | 26 | -0.57 | .574 |
| Intervention Group ($\gamma_{01}$) | -13.74 | 8.63 | 24 | -1.59 | .124 |
| Week 1 or Week 2 × Intervention Group ($\gamma_{11}$) | 3.81 | 5.33 | 26 | 0.71 | .481 |
| BMI ($\gamma_{02}$) | 4.78 | 1.78 | 24 | 2.69 | .013^*^ |
| Sex ($\gamma_{03}$) | -36.48 | 5.69 | 24 | -6.41 | <.001^***^ |

Note. Week 1 or Week 2 was coded as a dummy variable (Week 1 = 0, Week 2 = 1). Intervention Group was coded as a dummy variable (UIC = 0, PIC = 1). Standard Error (SE). Degree of Freedom (*df*). ^*^ *P*<.05 ^**^ *P*<.01 ^***^ *P*<.001

Table S2-6. The change in physical activity from Week 1 without intervention to Week 2 with intervention as affected by differences in intervention criteria (Model Set B) (Steps)

|  | Coefficient | SE | *df* | *t* | *P* |
| --- | --- | --- | --- | --- | --- |
| Intercept ($\gamma_{00}$) | 256.42 | 435.79 | 24 | 0.59 | .562 |
| Week 1 or Week 2 ($\gamma_{10}$) | 25.90 | 45.22 | 26 | 0.57 | .572 |
| Intervention Group ($\gamma_{01}$) | -67.40 | 94.79 | 24 | -0.71 | .484 |
| Week 1 or Week 2 × Intervention Group ($\gamma_{11}$) | 2.09 | 63.95 | 26 | 0.03 | .974 |
| BMI ($\gamma_{02}$) | 18.09 | 22.78 | 24 | 0.79 | .435 |
| Sex ($\gamma_{03}$) | -118.83 | 72.94 | 24 | -1.63 | .116 |

Note. Week 1 or Week 2 was coded as a dummy variable (Week 1 = 0, Week 2 = 1). Intervention Group was coded as a dummy variable (UIC = 0, PIC = 1). Standard Error (SE). Degree of Freedom (*df*). ^*^ *P*<.05 ^**^ *P*<.01 ^***^ *P*<.001

Table S2-7. The change in physical activity from Week 1 without intervention to Week 2 with intervention as affected by differences in intervention criteria (Model Set B) (Distance moved)

|  | Coefficient | SE | *df* | *t* | *P* |
| --- | --- | --- | --- | --- | --- |
| Intercept ($\gamma_{00}$) | 128.86 | 195.66 | 24 | 0.66 | .516 |
| Week 1 or Week 2 ($\gamma_{10}$) | 11.83 | 19.36 | 26 | 0.61 | .547 |
| Intervention Group ($\gamma_{01}$) | -30.72 | 42.15 | 24 | -0.73 | .473 |
| Week 1 or Week 2 × Intervention Group ($\gamma_{11}$) | 3.72 | 27.37 | 26 | 0.14 | .893 |
| BMI ($\gamma_{02}$) | 7.35 | 10.23 | 24 | 0.72 | .479 |
| Sex ($\gamma_{03}$) | -68.47 | 32.76 | 24 | -2.09 | .047^*^ |

Note. Week 1 or Week 2 was coded as a dummy variable (Week 1 = 0, Week 2 = 1). Intervention Group was coded as a dummy variable (UIC = 0, PIC = 1). Standard Error (SE). Degree of Freedom (*df*). ^*^ *P*<.05 ^**^ *P*<.01 ^***^ *P*<.001
